# Towards phenotyping adaptive traits in camels: A study of the influence of hypotonic saline solutions on blood cell area

**DOI:** 10.1371/journal.pone.0298336

**Published:** 2024-03-11

**Authors:** Hasan Alhaddad, Aisha Alnughaimish, Dalal Alhajeri, Bader H. Alhajeri

**Affiliations:** Department of Biological Sciences, Faculty of Science, Kuwait University, Shadadiya, Kuwait; Cairo University Faculty of Veterinary Medicine, EGYPT

## Abstract

Single-humped camels are livestock of physical, physiological, and biochemical adaptations to hot desert environments and to water scarcity. The tolerance of camels to water deprivation and their exceptional capacity for rapid rehydration requires blood cells with membranes of specialized organization and chemical composition. The objectives of this study are to examine the changes in the area (a proxy for volume) of camel blood cells in solutions with decreasing concentrations of NaCl and consequently identify the conditions under which blood cells can be phenotyped in a large population. Whole-blood samples from three healthy adult female camels were treated with four different concentrations of NaCl and examined at six incubation-periods. Observationally, red blood cells in all treatments remained intact and maintained their elliptical shape while white blood cells experienced some damage, lysing at concentrations below 0.90%. Average basal (in 0.90% NaCl) RBC area was ~15 μm^²^ and swelled in the various treatments, in some cases reaching twice its original size. Excluding the damaged cells, the average area of combined WBCs, ~32.7 μm^²^, expanded approximately three times its original size. We find that camel WBCs, like their RBCs, are adapted to hypotonic environments, and are capable of expanding while maintaining their structural integrity.

## Background

The dromedary camel, *Camelus dromedarius*, is a hallmark of hot deserts, being tolerant of high temperatures, and being able to conserve water efficiently [1, 2]. Camel blood plays a central role in these unique adaptations [3, 4]. Camel blood is similar to that of other mammals, having similar blood cell types, biochemical composition, and general function [5, 6]. Nonetheless, camels’ blood cells, particularly red blood cells (RBCs), differ from most mammals in its shape, size, and ability to swell extensively in hypotonic environments [4, 7, 8].

The erythrocytes of most mammals are round, thick, and biconcave, while those of camelids (e.g., dromedary, llama) are elliptical in shape, thin, and flat [5, 9]. Camel WBCs have not received as much attention as their RBCs, likely due to their similarity in shape/size to that of other mammals, thus apparently lacking adaptive value [6]. The previously reported dimensions and area of camel RBCs have been inconsistent (for summary see [8]), likely due to the camels’ individual variation and their hydration status, along with differences in measuring methods. The dimensions of camels’ RBCs across studies averaged 7.8 μm in length (i.e., long axis), 4.6 μm in width (i.e., short axis), and 0.95 μm in thickness [8, 10]. Using these reported measurements of camel RBCs, their area should roughly average 28.7 μm^²^ (range 18.7–42.8 μm^²^).

Veterinary hematological parameters are frequently reported for camels from different populations and locations [6, 11–14]. Camel RBCs are frequently studied for their adaptive value, as they exhibit extended survival and flexible shape. During the summer, when the camel is enduring the hot weather and undergoing prolonged dehydration, their RBCs stay alive for a long time [15] in a shrunken state, while still maintaining their ellipsoid shape [3, 4]. When it rehydrates rapidly, converting the blood stream to a hypotonic environment, their RBCs do not lyse, and just swell and get rounder in shape [4, 7]. This suggests that camels mainly store water in their RBCs (and other cells), rather than in their fat deposits (e.g., hump) or in their specialized stomach compartments [16–18].

The aforementioned camel adaptations have been attributed to the composition and location of lipids and proteins in camel RBC membranes are heterogenous [19]. The membranes of camel RBCs have an unusual protein to lipid (w/w) ratio (around 3) compared to the RBCs of humans (around 1.25). Therefore, the shape, stability, and elasticity of the RBCs of the camel are likely due to their lipoprotein content, particularly the cytoskeletal protein, spectrin [19–21].

Apparently, not all camel RBCs are equally adapted to aridity, given the wide differences in the reported RBC measurements (see above), and the fact that camels vary in their water requirements. The latter is particularly evident among camel breeds associated with the Arabian Peninsula. For example, the “Aljody” camel breed of Iraq (also known as “Judi”, “Jowada”, and “Jowdeyat”) [22, 23] is sensitive to water shortage and needs to drink regularly. Ingham [24] notes that these camels are kept by their breeders near rivers during the summer, while other breeds are usually kept in the desert interior away from such water sources.

To investigate the genetic basis of the adaptation of camel RBCs, and likely WBCs, to aridity, a phenotyping protocol of the RBCs’ response (de)hydration needs to be established. This can then be used to phenotypically screen different camels and conduct candidate gene sequencing, association, or whole genome sequencing studies [25]. Here, our objectives are to (1) examine the changes in the area (a proxy for volume) of camel blood cells (RBCs and WBCs) in response to solutions with decreasing concentrations of NaCl solutions, and to (2) identify the conditions (NaCl concentration and incubation time) under which these blood cells, especially RBCs, can be phenotyped in a large population in an efficient manner.

## Materials and methods

### Animals

All animal handling and sampling procedures have been approved by the Ethics Committee for the Use of Laboratory Animals (ECULA), Kuwait University (code: DBS/IRB(ECULA)19-007).

Three healthy adult half-sibling female camels (Cdrom 239, 243, 245, Shaele camel-type [25]), raised under identical environmental conditions, diet, and access to water were selected as blood sample sources. During routine veterinary examination, whole-blood samples (6 ml) were withdrawn from their jugular veins directly into sterile EDTA tubes, which were then inverted 2–3 times, placed on an ice box for transportation, and then stored in 4ºC for ~ 24 hours.

### Treatments

Each of the three whole-blood samples were aliquoted (1 ml) into four tubes each of which was mixed with an equal volume of NaCl solution. Four decreasing concentrations of NaCl (0.90, 0.75, 0.50, and 0.25%) were used (3 camel blood samples x 4 NaCl concentrations = 12 tubes). The 0.9% NaCl treatment was used as a surrogate of the natural state of the blood stream of hydrated camels (i.e., control) and to which the other treatments were compared to. The solutions were mixed by inverting 2–3 times and then placed on a slow shaker. Aliquots (~20 μl) were sampled from each tube at six incubation periods (0.25, 0.5, 1, 2, 4, and 24 hours).

### Slide preparation

Thin blood film slides were prepared for each of the three camel samples in the four solutions at each of the six incubation periods using ~20 μl of solution. Blood films were prepared using a clean spreader slide that was held at a 45° angle toward the drop of blood on the specimen slide. The sample spread along the edge of the slide by capillary action. The spreader slide was then pushed forward rapidly. The slides were then dried and fixed in methanol (for three minutes) to preserve the morphology of cells. Cells were then stained using Wright’s stain (Sigma-Aldrich, USA) for ~12 min then rinsed with distilled water. Cover slips were mounted over the blood films using DPX mountant and placed on a hot plate for two minutes. The blood film slides were examined under a compound light microscope (CH30RF200, Olympus Optical, Japan) at 100X power oil immersion. The blood film slides were imaged using the Leica DM500 Digital Microscope (Leica, Germany) at the Nanoscopy Center of Kuwait University.

### Area calculation

The RBCs and the WBCs were observed using a compound light microscope at 100x, and 25 RBCs from random locations on the slide were measured (in μm) in their long (“L”–the longest distance of the oval shape) and short axes (“W”–the longest distance of the oval shape perpendicular to “L”) [4]. Similarly, 25 randomly selected WBCs were characterized and measured (in μm) along their diameter (“D”–the diameter of the cell). WBCs were categorized based on their general morphology into basophils, eosinophils, lymphocytes, monocytes, and neutrophils. The area of RBCs was approximated using the following equation $A=\pi \frac{L}{2}*\frac{W}{2}$ and WBCs using *A* = *πr*^2^ (“r”–the radius of the cell, D/2).

### Statistical analysis

All the following analyses were conducted in R [26]. Unless otherwise stated, analyses used the base R library. Also, for all the following analyses, typical and atypical lymphocytes (white blood cell types) were combined, and all damaged cells (mostly WBCs) were removed from the dataset prior to analysis. Although blood was collected (in equal proportions) from three different camels (see above), preliminary analyses indicated no significant effect of camel identity on cell area, and this factor did not interact with any of the other factors considered in the analyses below, and as such camel identity was not considered further in subsequent analyses (i.e., data was combined from all three different camels).

In our first set of analyses, we used a multifactorial Analysis of Variance (ANOVA) framework to determine the influence of time (‘Time’), NaCl concentration (‘Conc’), cell-type (‘Cell’), and their interactions on cell size (’Area’). Although continuous, the numerical predictor variables ‘Time’ and ‘Conc’ are measured at discrete intervals, and thus resemble discrete variables. These variables were treated as categorical predictors (i.e., factors) rather than continuous predictors (i.e., covariates) as their few values (i.e., six levels for ‘Time’ and four levels for ‘Conc’) (Table 1) would lead to poor regression estimates if they are treated as continuous predictors. Furthermore, treating these variables as categorical allows for testing their influence on ‘Area’ even in cases where their relationships are nonlinear (i.e., no linear relationship assumption is made).

**Table 1:** Descriptive summary of the areas of camel blood cells incubated in NaCl solutions. Mean (±SD) areas (in μm^²^) of blood cells incubated in four different NaCl concentration.

Cell Type	Conc. (NaCl)^1^	No. cells	Mean Area (μm^²^)
RBC	0.25%	450	29.76±5.81
	0.50%	450	27.05±5.84
	0.75%	450	21.77±5.55
	0.90%	450	14.87±3.18
WBC	0.25%	291	90.81±42.04
	0.50%	306	50.48±27.45
	0.75%	366	43.13±23.00
	0.90%	447	32.69±14.30

^1^All incubation times were combined for each concentration.

Both parametric ANOVA assumptions were not satisfied in our dataset. A quantile-quantile plot and a Shapiro-Wilk test do not support normality of model residuals, and both Bartlett’s [27] and Levene’s tests do not support homogeneity of variance across groups for all factors, the latter implemented using the leveneTest() function in the *car* package [28]. These assumptions are similarly violated when ‘Area’ is log-transformed. As an alternative, we analyzed the data using the distribution‐free Permutational Multivariate Analysis of Variance (PERMANOVA) [29] using the adonis() function in the in the *vegan* library [30]. PERMANOVA tests differences in centroids (or in the case of univariate data like ours, the difference in means) using an ANOVA-like design to test the response of one or more variables to one or more factors but obtains *p*-values for each model term via permutation [29]. Because our data are not normally distributed, the *F* ratio calculated by PERMANOVA is a pseudo-*F* statistic [31]. This method does not assume normality of residuals [29], but is sensitive to heterogeneity in dispersions (or variance in the case of univariate data like ours) for unbalanced designs [32]. Since our data is not exactly balanced (although it is close to being balanced) (Table 1), significant *p*-values could be driven by the unequal variance across the groups. Our previous analyses indicated heterogeneous variance across groups for all factors (see above), and as such the results of the PERMANOVA may not be conservative. As such, PERMANOVA results were verified both visually using scatterplots (see below) and via post-hoc pairwise PERMANOVA comparisons at the level of single factors; the latter also serves to determine which pairs of levels significantly differ from each other.

For significant interaction terms, pairwise comparisons were conducted within individual levels of the factors. These pairwise PERMANOVAs were carried out using the adonis.pair() function in the *EcolUtils* library [33]. In these pairwise analyses, we used Holm’s [34] method to correct for multiple comparisons, which maintains a table wide error rate of 5%.

For all the PERMANOVAs, we computed Euclidean distances among the samples based on a single response variable (‘Area’) and computed *p*-values using 999 permutations in *vegan*. The amount of the total variation explained by each model term (each factor and their interactions) was calculated by *vegan* as the ratio of the SS of each factor to the total SS of the model (i.e., *R*^2^). The significance level (*p*) was set at α = 0.050 for all analyses.

## Results

### General cell morphology, survival, and area

**Fig 1:**
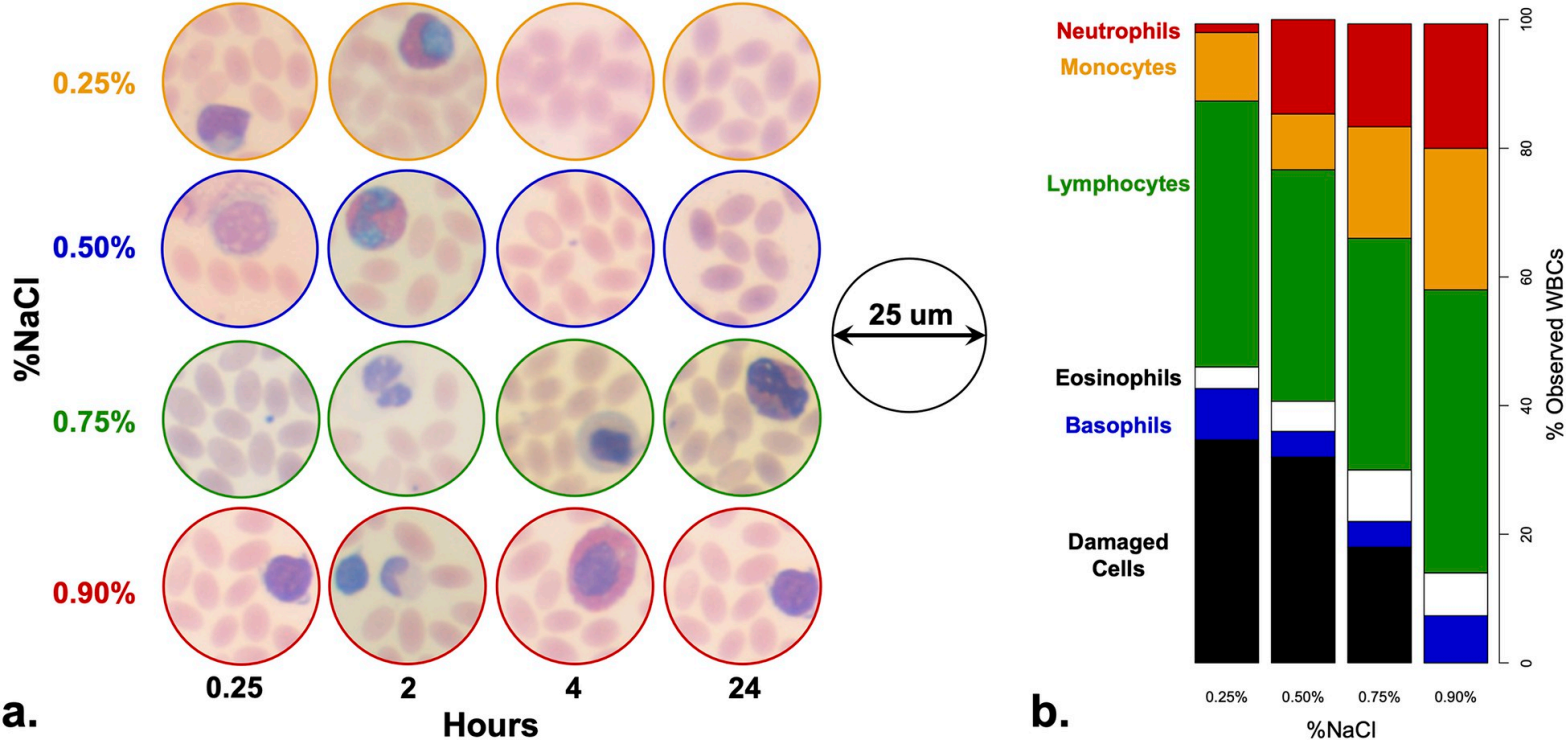
Camel blood cells treated with different concentrations of NaCl. a. microscopic images of camel-blood cells treated with different NaCl solutions. Unnucleated ellipsoid cells correspond to RBCs whereas nucleated cells represent WBCs. Cells were observed at 100x magnification and the diameter of the circular image is 25 μm. b. proportions of examined WBCs based on cell-type in four NaCl treatments. Due to the different levels of observed cell damage, the total number of examined WBCs in 0.25, 0.5, 0.75, and 0.9% NaCl concentrations were 291, 306, 366, and 447, respectively (Table 1). Damaged WBCs were observed in low concentration of NaCl (0.25% NaCl = 34.7%, 0.5% NaCl = 32%, and 0.75% NaCl = 18%).

The RBCs were unnucleated and elliptically shaped with no apparent damage across the four different NaCl concentrations (Fig 1a). On the other hand, damaged WBCs were observed only in NaCl concentrations <0.90%. The proportions of damaged WBCs were approximately 18, 32, and 34.7% in the 0.75, 0.50, and 0.25% NaCl concentrations, respectively (Fig 1b, S1 Table). The observed WBC damage was in the form of complete nuclear loss or unidentifiable nuclear shape which was visible as early as after 15 minutes of incubation in 0.50% NaCl solution and after four hours incubation in 0.75% NaCl solution. The non-damaged WBCs exhibited moderate swelling with clearly visible nuclei (Fig 1a). Lymphocytes displayed moderate nuclei shrinkage and minor cytoplasmic vacuolation (i.e., atypical lymphocytes) and appeared in the form of an irregular nuclear contour, amoeboid nuclear shape, or nuclear clefts.

**Fig 2:**
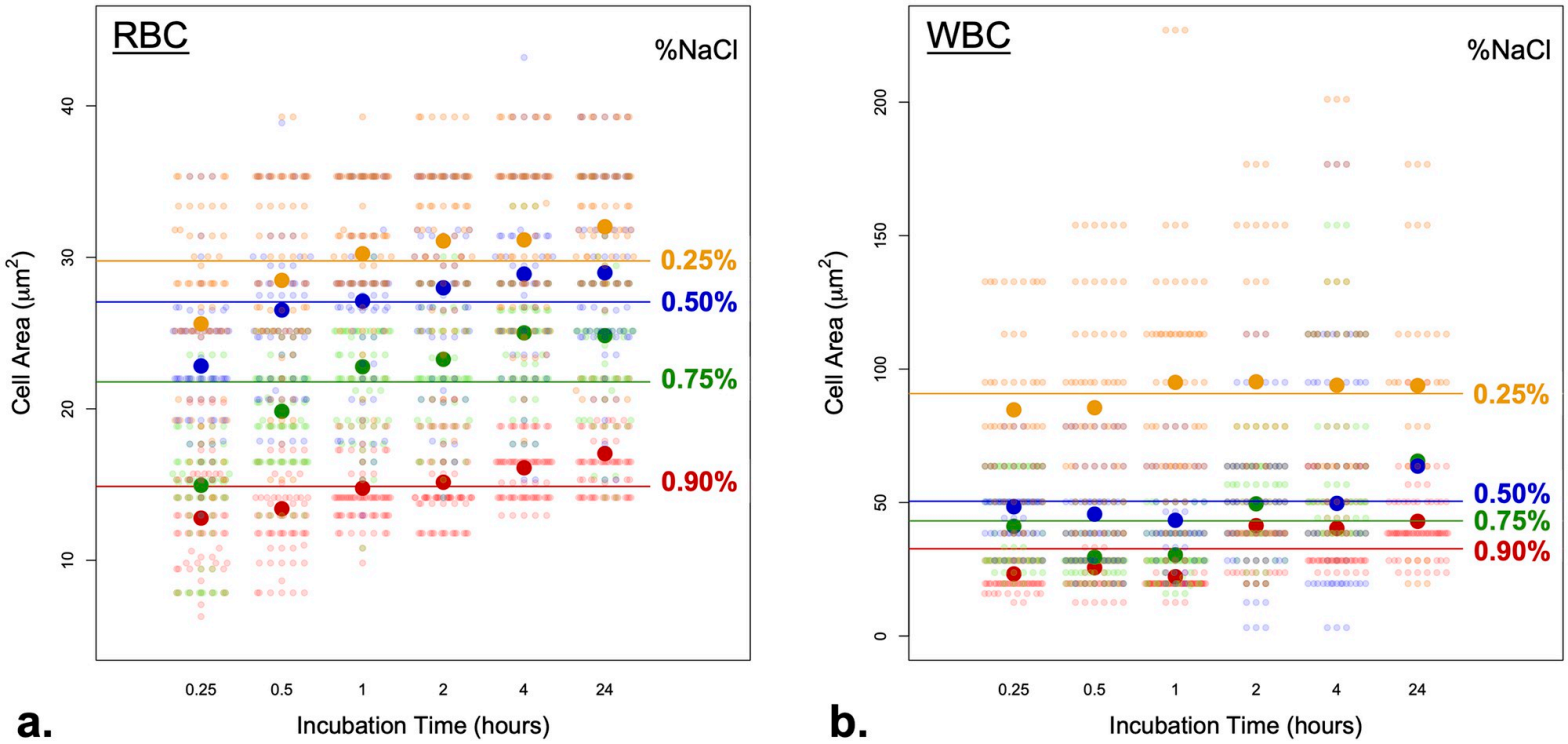
Camel blood cell area and its relation to NaCl concentrations and incubation times. a. Changes in RBCs’ area in four NaCl solutions (0.25, 0.50, 0.75, and 0.90% NaCl) and in six incubation times (0.25, 0.5, 1, 2, 4, and 24 hrs). Area measurements are colored (red, green, blue, and orange) according to the NaCl concentration. Small circles represent an area measurement of an individual cell whereas large circles represent the mean area size at a specific incubation time. The horizontal-colored lines represent the mean cell area in different NaCl concentrations. b. Changes in WBCs area in response to different NaCl concentrations and incubation times. Plot details are identical to those found in (a.). Note that the y-axis of the RBCs in (a) and of the WBCs in (b) are not identical, and that WBCs have larger areas as expected.

Using the 0.90% NaCl concentration as a reference to determine typical cell area, RBCs and WBCs varied greatly in their area, with a mean (±SD) of 14.87±3.18 μm^²^ for RBCs (Fig 2a–red horizontal line) and 32.69±14.30 μm^²^ for WBCs (all types combined) (Table 1, Fig 2b–red horizontal line). The variation in the RBCs’ area in each concentration was lower than that of the WBCs, likely due to the fact that the differently-shaped WBC types were pooled together in the analysis. As a qualitative comparison of the expansion of the blood cells’ area, we compared the mean area of blood cells in 0.90% NaCl to the mean area in 0.25% NaCl (Fig 2). The RBCs’ area increased around two folds to reach a maximum mean area (±SD) of 29.76±5.81 μm^²^; the WBCs increased ~2.77 folds to a maximum mean area (±SD) of 90.81±42.04 μm^²^ (Table 1). With regard to the incubation time, we observed that the changes in cell area (i.e., increase in area), especially for RBCs, were minimal after 30 minutes of incubation in all concentrations of NaCl (Fig 2a). In other words, 30 minutes of incubation resulted in the largest change in cell area after which little change was observed (e.g., 30 minutes vs. 1 hour).

### Contributors to cell area changes

First, we examined the effects of three factors: (1) incubation time, (2) NaCl concentration, and (3) cell type (i.e., RBC or WBC) on the measured cell area. According to the overall PERMANOVA, all three factors had a significant effect on cell area (all *p* < 0.050; Table 2). However, while significant, incubation time explained only 1.48% of the total variation in cell area (*R*^2^ = 0.0148), while the other two terms, NaCl concentration and cell type, explained a much larger proportion of the variation (*R*^2^ = 0.1646 and 0.2986 respectively; Table 2). All interaction terms in the model were significant (all *p* < 0.050; Table 2). However, all interactions were of low effect size (*R*^2^ ≤ 0.0128), except for the two-way interaction between NaCl concentration and cell-type (*R*^2^ = 0.0973; Table 2), and thus only this interaction term was further examined. Given that all interactions are significant, the results of the main effects should be interpreted (if at all) with caution. Furthermore, the effect of NaCl concentration on cell area was examined for each cell type separately (see below).

**Table 2:** Overall PERMANOVA testing the effect of incubation time (‘Time’), NaCl concentration (‘Conc’), cell type (‘Cell’), and their interactions on cell size (area).

	df	SS	MS	*F*	*R* ^2^	*p*
Time	5	35270	7054	23.74	0.0148	0.001
Conc	3	392137	130712	439.98	0.1646	0.001
Cell	1	711352	711352	2394.41	0.2986	0.001
Time × conc	15	30512	2034	6.85	0.0128	0.001
Time × cell	5	23917	4783	16.10	0.0100	0.001
Conc × cell	3	231860	77287	260.15	0.0973	0.001
Time × conc × cell	13	17683	1360	4.58	0.0074	0.001
Residuals	3164	939988	297		0.3945	
Total	3209	2382720			1.0000	

Notes: df = degrees of freedom; SS = Type-I (sequential) sums of squares; MS = mean squares; *F* = pseudo-*F* statistic; *R*^2^ = amount of the total variation explained by each model term (term SS/total SS); *p* = *p-*value based on 999 permutations. All model terms are significant (*p*<0.050). As factors are unbalanced, the main effects should be interpreted with caution, as they depend on the order of the terms in the model. In this case, main effects should not be interpreted given the significant interactions (see Results). For WBCs, within the level of ‘Time’ = 2, there are 3 levels of ‘Conc’ (0.25, 0.75, 0.9 –level 0.5 is missing), and within the level of ‘Time’ = 4, there are 3 levels of ‘Conc’ = 0.25, 0.5, 0.9 –level 0.75 is missing). Alternatively, for WBCs, within the level of ‘Conc’ = 0.5, there are 5 levels of ‘Time’ (0.25, 0.5, 1, 4, 24 –level 2 is missing), and within the level of ‘Conc’ = 0.75, there are 5 levels of ‘Time’ (0.25, 0.5, 1, 2, 24 –level 4 is missing). See S1 Table for more information.

### Effect of incubation time on cell area

For the RBCs, both incubation time, NaCl concentration, and their interaction had a significant effect on cell area (all *p* < 0.050; Table 3). While significant, the interaction effect was extremely small (*R*^2^ = 0.0120) and as such this effect was not considered further, especially considering that the main effect of incubation time was much smaller than NaCl concentration (*R*^2^ = 0.0863 and 0.5430 respectively; Table 3). Similar results were found for the WBCs, with both incubation time, NaCl concentration, and their interaction having a significant effect on cell area (all *p* < 0.050), but with the effect size being much larger for NaCl concentration (*R*^2^ = 0.4061) than for incubation time (*R*^2^ = 0.0327) and its interaction with NaCl concentration (*R*^2^ = 0.0183; Table 3).

**Table 3:** PERMANOVA testing the effect of incubation time (‘Time’) and NaCl concentration (‘Conc’), and their interactions on cell size (area).

Cell		df	SS	MS	*F*	*R* ^2^	*p*
RBC	Time	5	9237	1847.5	85.46	0.0863	0.001
	Conc	3	58130	19376.7	896.37	0.5430	0.001
	Time × conc	15	1287	85.8	3.97	0.0120	0.001
	Residuals	1776	38391	21.6		0.3586	
	Total	1799	107046			1.0000	
WBC	Time	5	54218	10844	16.69	0.0327	0.001
	Conc	3	674421	224807	346.09	0.4061	0.001
	Time × conc	13	30417	2340	3.60	0.0183	0.001
	Residuals	1388	901597	650		0.5429	
	Total	1409	1660653			1.0000	

Notes: all column labels correspond to those in Table 2. All model terms are significant (*p*<0.050). For WBCs, within the level of ‘Time’ = 2, there are 3 levels of ‘Conc’ (0.25, 0.75, 0.9 –level 0.5 is missing), and within the level of ‘Time’ = 4, there are 3 levels of ‘Conc’ = 0.25, 0.5, 0.9 –level 0.75 is missing). Alternatively, for WBCs, within the level of ‘Conc’ = 0.5, there are 5 levels of ‘Time’ (0.25, 0.5, 1, 4, 24 –level 2 is missing), and within the level of ‘Conc’ = 0.75, there are 5 levels of ‘Time’ (0.25, 0.5, 1, 2, 24 –level 4 is missing). See S1 Table for more information.

### Effect of concentration on cell area

Based on the *R*^2^ values, the effect of NaCl concentration on cell size was slightly greater for RBCs (*R*^2^ = 0.5430; Table 3, Fig 2a) than for WBCs (*R*^2^ = 0.4061; Table 3, Fig 2b). Post hoc pairwise PERMANOVAs indicated that all pairs of NaCl concentrations were significantly different both in RBCs and in WBCs (all *p* < 0.050), even after Holm correction (all Holm-*p* < 0.050; Table 4). Among the three neighboring NaCl concentration pairs, the effect was strongest between 0.75% NaCl and 0.90% NaCl (*R*^2^ = 0.3684) and weakest between 0.25% NaCl and 0.50% NaCl (*R*^2^ = 0.0513; Table 4, Fig 2a–see difference in neighboring horizontal lines) in RBCs. A different trend was found for WBCs, where among the three neighboring NaCl concentration pairs, the effect was strongest between 0.25% NaCl and 0.50% NaCl (*R*^2^ = 0.2463) and weakest between 0.50% NaCl and 0.75% NaCl (*R*^2^ = 0.0208; Table 4, Fig 2b—see the difference in the neighboring horizontal lines).

**Table 4:** Pairwise PERMANOVAs testing the effect of NaCl concentration (‘Conc’) on cell size (area), divided by cell type (RBC vs. WBC).

Cell	‘Conc’ pair	SS	MS	F	R^2^	*p*	Holm-*p*
RBC	*0*.*25 <-> 0*.*50*	*1652*.*2*	*1652*.*2*	*48*.*60*	*0*.*0513*	*0*.*001*	*0*.*006*
	0.25 <-> 0.75	14350.3	14350.3	443.74	0.3307	0.001	0.006
	0.25 <-> 0.90	49887.1	49887.1	2269.52	0.7165	0.001	0.006
	*0*.*50 <-> 0*.*75*	*6263*.*8*	*6263*.*8*	*192*.*79*	*0*.*1767*	*0*.*001*	*0*.*006*
	0.50 <-> 0.90	33381.4	33381.4	1508.25	0.6268	0.001	0.006
	*0*.*75 <-> 0*.*90*	*10724*.*9*	*10724*.*9*	*523*.*72*	*0*.*3684*	*0*.*001*	*0*.*006*
WBC	*0*.*25 <-> 0*.*50*	*242643*.*0*	*242643*.*0*	*194*.*44*	*0*.*2463*	*0*.*001*	*0*.*006*
	0.25 <-> 0.75	368571.0	368571.0	342.07	0.3431	0.001	0.006
	0.25 <-> 0.90	595455.0	595455.0	725.76	0.4965	0.001	0.006
	*0*.*50 <-> 0*.*75*	*9000*.*6*	*9000*.*6*	*14*.*25*	*0*.*0208*	*0*.*001*	*0*.*006*
	0.50 <-> 0.90	57491.0	57491.0	134.45	0.1518	0.001	0.006
	*0*.*75 <-> 0*.*90*	*21939*.*0*	*21939*.*0*	*62*.*56*	*0*.*0716*	*0*.*001*	*0*.*006*

Notes: ‘Conc’ pair = pair of NaCl concentrations being compared; *p* = raw *p-*value based on 999 permutations; Holm-*p* = *p* value corrected for multiple comparisons using Holm’s (1979) method. All other column labels correspond to those in Table 2. All pairs significantly differ at *p*<0.050. Neighboring ‘Conc’ pairs are in italics.

When dividing the dataset by the different NaCl concentrations, for 0.25% NaCl concentration, both incubation time and cell type had a significant effect on cell size (all *p* < 0.050), but not their interaction (*p* = 0.857; Table 5). The effect size was much greater for cell type (*R*^2^ = 0.5502) than for incubation time (*R*^2^ = 0.0107; Table 5). Similar results were found for the other NaCl concentrations (Table 5 –see S1 File). Based on the *R*^2^ values, the effect of cell type on cell area within the NaCl concentration levels was greatest for 0.25% NaCl (*R*^2^ = 0.5502), followed respectively by 0.90% NaCl (*R*^2^ = 0.4244), 0.75% NaCl (*R*^2^ = 0.3006), and finally 0.50% NaCl (*R*^2^ = 0.2693).

**Table 5:** PERMANOVA testing the effect of incubation time (‘Time’), cell type (‘Cell’), and their interactions on cell size (area) for the four NaCl concentrations.

% NaCl		df	SS	MS	*F*	*R* ^2^	*p*
0.25	Time	5	12731	2546	3.57	0.0107	**0.005**
	Cell	1	652841	652841	916.25	0.5502	**0.001**
	Time × cell	5	1463	293	0.41	0.0012	0.857
	Residuals	729	519422	713		0.4377	
	Total	740	1186458			1.0000	
0.50	Time	5	17862	3572	11.71	0.0517	**0.001**
	Cell	1	92958	92958	304.70	0.2693	**0.001**
	Time × cell	4	7069	1767	5.79	0.0204	**0.001**
	Residuals	745	227278	305		0.6584	
	Total	755	345167			1.0000	
0.75	Time	5	46547	9309	55.60	0.1556	**0.001**
	Cell	1	89909	89909	536.99	0.3006	**0.001**
	Time × cell	4	27790	6947	41.49	0.0929	**0.001**
	Residuals	805	134783	167		0.4507	
	Total	815	299029			1.0000	
0.90	Time	5	23774	4755	71.92	0.1423	**0.001**
	Cell	1	70878	70878	1072.18	0.4244	**0.001**
	Time × cell	5	13829	2766	41.84	0.0828	**0.001**
	Residuals	885	58505	66		0.3503	
	Total	896	166986			1.0000	

Notes: all column labels correspond to those in Table 2. Significant model terms (*p*<0.050) are in bold. For WBCs, within the level of ‘Time’ = 2, there are 3 levels of ‘Conc’ (0.25, 0.75, 0.9 –level 0.5 is missing), and within the level of ‘Time’ = 4, there are 3 levels of ‘Conc’ = 0.25, 0.5, 0.9 –level 0.75 is missing). Alternatively, for WBCs, within the level of ‘Conc’ = 0.5, there are 5 levels of ‘Time’ (0.25, 0.5, 1, 4, 24 –level 2 is missing), and within the level of ‘Conc’ = 0.75, there are 5 levels of ‘Time’ (0.25, 0.5, 1, 2, 24 –level 4 is missing). See S1 Table for more information.

## Discussion

The dromedary camel is a desert icon. It has numerous physical and physiological adaptations to this environment [35]. Even their blood cells, especially the RBCs, are adapted to this environment, with their unique shape, membrane composition, which allows for an adapted response to hyper- and hypotonicity [7, 19, 20]. Camel RBCs (and those of other camelids, such as llama) are ellipsoid in shape and flat, compared to the round and concaved RBCs of most other mammals [9]. Camels’ RBCs differ from those of other mammals in structure and physiology which adapts them to periods of prolonged dehydration and allows them to rapidly rehydrate when water is available without any adverse effects (as would be incurred on less adapted mammals). When dehydrated, camel RBCs shrink without changing their ellipsoid shape; after rapid rehydration, RBCs swell and change shape (to a sphere) without lysing [9].

The most researched camel adaptations are physiological and were based on comparisons to other mammals, including humans and cattle [7]. To investigate camel blood cell adaptation to aridity at a molecular level and to identify the genes associated with it, several requirements need to be fulfilled. These include (1) developing a phenotyping protocol for camel blood cells, (2) applying the developed protocol on a diverse sample of camels from breeds with different dehydration tolerance (e.g., Aljoudy vs. Majaheem), and (3) using the detected intraspecific variation in dehydration adaptation to identify associated genes using genetic (e.g., candidate gene sequencing), genomic (e.g., genome-wide association studies (GWAS) or whole genome sequencing), or proteomic tools (e.g., lipoprotein profiles). Our current study focused on the first requirement: developing a phenotyping protocol. Most previous studies of the adaptability of camel blood cells focused on RBCs [4, 7, 8]. In addition to RBCs, our study also included WBCs.

The camels’ RBCs did not lyse even at the lowest NaCl concentration (0.25%) and for the longest incubation period. This is in agreement with previously reported observations [4, 7]. Our observation that camel WBCs are predominated by lymphocytes is also in agreement with previous reports [11]. When treated with NaCl solutions of below 0.90% concentration, up to ~35% of the cells were damaged (Fig 1b). The fact that nearly 65% of the WBCs survived incubation in the hypotonic solution (0.25% NaCl) suggests that some of the adaptive attributes of RBCs are shared with WBCs (e.g., resistance to lysis) [20]. WBCs’ resistance to lysis may explain the difficulty in extracting DNA from whole-blood samples and the inconsistent DNA quantities obtained from same volumes of starting samples [36]. In other words, standard DNA extraction protocols result in incomplete WBC lysis and produces inconsistent quantities of DNA.

If we consider incubation in 0.90% NaCl solution as the baseline of cell shape, both RBCs (i.e., ellipsoid shape) and WBCs (i.e., round shape) appeared normal. On average, the baseline area of the RBCs was 14.87±3.18 μm^²^, which is relatively smaller than observed in previous studies (summarized in [8]). We attribute this discrepancy to differences in starting conditions of the collected samples (i.e., season, hydration status, stress, etc.). Despite differences in the shape, the average RBC area of camels appears intermediate when compared to breeds of goat (9.50±0.96 μm^²^), Sheep (16.38±1.26 μm^²^), cattle (22.56±2.49 μm^²^), horses (27.93±3.42 μm^²^), and dog (38.83±4.30 μm^²^) [37].

As for the WBCs, the baseline area was 32.69±14.30 μm^²^; the large standard deviation is likely due to combining all WBC types into a single group. The response of RBCs to treatments in different NaCl concentrations was primarily affected by incubation time during the first 30 minutes of incubation, after which no significant area changes were observed (Fig 2a). On the contrary, WBCs showed no significant differences with regard to incubation times under all NaCl concentrations (Fig 2b). Therefore, it is advisable when phenotyping responses of RBCs to hypotonicity to incubate them in the solution for at least 30 minutes, whereas 15 minutes of incubation time is sufficient to phenotype WBCs.

To phenotype cell adaptation at a large scale, and to study its genetics, we recommend the following regarding sample collection and blood treatment. We recommend that blood samples should be collected (1) from adult female camels of known breed and location, (2) during the same season, preferably winter, and (3) from camels with unrestricted access to water and feed. Based on our current investigation and findings, we recommend (1) incubating whole-blood samples (RBCs and WBCs) in 0.90% NaCl solution to establish their baseline area size, (2) incubating whole-blood samples (RBCs and WBCs) in 0.25% NaCl solution to record the cell-area response to hypotonicity, (3) preparing and fixing slides after 30 minutes of incubation for RBCs and after 15 minutes of incubation for WBCs, and (4) measuring the dimensions of cells using the same method (e.g., light microscopy).

## Supporting information

S1 Table Microscopic count of individual blood cell-types incubated in different NaCl concentrations and for different periods. (XLSX)

S2 Table Dimension measurements and area calculation of cells used in the analysis. (XLSX)

S1 File Detailed additional supplementary results. (DOCX)
