# Supplementary material for: Towards phenotyping adaptive traits in camels: A study of the influence of hypotonic saline solutions on blood cell area
Source: PLoS One. 2024 Mar 11;19(3):e0298336. doi: 10.1371/journal.pone.0298336 (PMC10927079; doi:10.1371/journal.pone.0298336)
Supplement: S1 File — (DOCX) [file pone.0298336.s003.docx]

**Towards phenotyping adaptive traits in camels: a study of the response of blood cells to a hypotonic environment**

**Supplemental Results**

The relatively large interaction between ‘Conc’ and ‘Cell’ indicates that the effect of ‘Conc’ on ‘Area’ depends to a large degree on ‘Cell’ (and vice versa). To account for this interaction, individual follow-up PERMANOVAs (using the same setup as the overall PERMANOVA) were used to test the effect of ‘Conc’ on ‘Area’ separately for each level of ‘Cell’, as well as the effect of ‘Cell’ on ‘Area’ separately for each level of ‘Conc’. For both these analyses, the (minor) effect of ‘Time’ on ‘Area’ was accounted for by including it as the first term in the model (and thus its effect on ‘Area’ was removed prior to examining the effect of the other factors on ‘Area’). The results of these follow-up PERMANOVAs were similar to the overall PERMANOVA.

For 0.5 NaCl concentration, both ‘Time’, ‘Cell’, and their interaction had a significant effect on ‘Area’ (all *p* < 0.050), but with the effect size being much larger for ‘Cell’ (*R*2 = 0.2693) than for ‘Time’ (*R*2 = 0.0517) and its interaction with ‘Cell’ (*R*2 = 0.0204; **Table S1**).

**Table S1. PERMANOVA testing the effect of time (‘Time’) and cell-type (‘Cell’), and their interactions on cell size ('Area') for 0.5 NaCl concentration.**

|  | df | SS | MS | *F* | *R*^2^ | *p* |
| --- | --- | --- | --- | --- | --- | --- |
| Time | 5 | 17862 | 3572 | 11.71 | 0.0517 | 0.001 |
| Cell | 1 | 92958 | 92958 | 304.70 | 0.2693 | 0.001 |
| Time:Cell | 4 | 7069 | 1767 | 5.79 | 0.0204 | 0.001 |
| Residuals | 745 | 227278 | 305 |  | 0.6584 |  |
| Total | 755 | 345167 |  |  | 1.0000 |  |

Notes: df= degrees of freedom; SS= Type-I (sequential) sums of squares; MS= mean squares; *F*= pseudo-*F* statistic; *R*^2^= amount of the total variation explained by each model term (term SS/total SS); *p*= *p-*value based on 999 permutations. As factors are unbalanced, the main effects should be interpreted with caution, as they depend on the order of the terms in the model.

For 0.75 NaCl concentration, both ‘Time’, ‘Cell’, and their interaction had a significant effect on ‘Area’ (all *p* < 0.050), but with the effect size being larger for ‘Cell’ (*R*2 = 0.3006) than for ‘Time’ (*R*2 = 0.1556) and its interaction with ‘Cell’ (*R*2 = 0.0929; **Table S2**).

**Table S2. PERMANOVA testing the effect of time (‘Time’) and cell-type (‘Cell’), and their interactions on cell size ('Area') for 0.75 NaCl concentration.**

|  | df | SS | MS | *F* | *R*^2^ | *p* |
| --- | --- | --- | --- | --- | --- | --- |
| Time | 5 | 46547 | 9309 | 55.60 | 0.1556 | 0.001 |
| Cell | 1 | 89909 | 89909 | 536.99 | 0.3006 | 0.001 |
| Time:Cell | 4 | 27790 | 6947 | 41.49 | 0.0929 | 0.001 |
| Residuals | 805 | 134783 | 167 |  | 0.4507 |  |
| Total | 815 | 299029 |  |  | 1.0000 |  |

Notes: all column labels correspond to those in Table S1.

For 0.9 NaCl concentration, both ‘Time’, ‘Cell’, and their interaction had a significant effect on ‘Area’ (all *p* < 0.050), but with the effect size being larger for ‘Cell’ (*R*2 = 0.4244) than for ‘Time’ (*R*2 = 0.1423) and its interaction with ‘Cell’ (*R*2 = 0.0828; **Table S3**).

**Table S3. PERMANOVA testing the effect of time (‘Time’) and cell-type (‘Cell’), and their interactions on cell size ('Area') for 0.9 NaCl concentration.**

|  | df | SS | MS | *F* | *R*^2^ | *p* |
| --- | --- | --- | --- | --- | --- | --- |
| Time | 5 | 23774 | 4755 | 71.92 | 0.1423 | 0.001 |
| Cell | 1 | 70878 | 70878 | 1072.18 | 0.4244 | 0.001 |
| Time:Cell | 5 | 13829 | 2766 | 41.84 | 0.0828 | 0.001 |
| Residuals | 885 | 58505 | 66 |  | 0.3503 |  |
| Total | 896 | 166986 |  |  | 1.0000 |  |

Notes: all column labels correspond to those in Table S1.

According to the WBC-only PERMANOVA, ‘Time’, ‘Conc’, and ‘CellType’, and their interactions all had a significant effect on ‘Area’ (all *p* < 0.050; **Table S4**). The three-way interaction between the factors (Time:Conc:CellType) was of moderate effect size (*R*2 = 0.0517; **Table S4**), and thus the main effects were not further interpreted in this model, and the effect of ‘Conc’ on ‘Area’ was examined for each ‘CellType’ separately (and vice versa). To account for this interaction, individual follow-up PERMANOVAs were used to test the effect of ‘Conc’ on ‘Area’ separately for each level of ‘CellType’, as well as the effect of ‘CellType’ on ‘Area’ separately for each level of ‘Conc’. For both these analyses, the (minor) effect of ‘Time’ on ‘Area’ was accounted for by including it as the first term in the model (and thus its effect on ‘Area’ was removed prior to examining the effect of the other factors on ‘Area’).

**Table S4. WBC-only PERMANOVA testing the effect of time (‘Time’), NaCl concentration (‘Conc’), WBC cell-type (‘CellType’), and their interactions on cell size ('Area').**

|  | df | SS | MS | *F* | *R*^2^ | *p* |
| --- | --- | --- | --- | --- | --- | --- |
| Time | 5 | 54218 | 10844 | 20.16 | 0.0326 | 0.001 |
| Conc | 3 | 674421 | 224807 | 418.02 | 0.4061 | 0.001 |
| CellType | 4 | 18465 | 4616 | 8.58 | 0.0111 | 0.001 |
| Time:Conc | 13 | 29214 | 2247 | 4.18 | 0.0175 | 0.001 |
| Time:CellType | 20 | 45004 | 2250 | 4.18 | 0.0271 | 0.001 |
| Conc:CellType | 12 | 46266 | 3856 | 7.17 | 0.0278 | 0.001 |
| Time:Conc:CellType | 37 | 85877 | 2321 | 4.32 | 0.0517 | 0.001 |
| Residuals | 1315 | 707189 | 538 |  | 0.4258 |  |
| Total | 1409 | 1660653 |  |  | 1.0000 |  |

Notes: all column labels correspond to those in Table S1.

The results of these follow-up PERMANOVAs were similar to the overall PERMANOVA. For Basophils, both ‘Time’, ‘Conc’, and their interaction had a significant effect on ‘Area’ (all *p* < 0.050), with the effect size being greater for ‘Conc’ (*R*2 = 0.4308) than for ‘Time’ (*R*2 = 0.1089) and its interaction with ‘Conc’ (*R*2 = 0.2456; **Table S5**). It is worth mentioning here that for this cell-type, time seems to strongly interact with concentration.

**Table S5. PERMANOVA testing the effect of time (‘Time’) and NaCl concentration (‘Conc’), and their interactions on Basophil cell size ('Area').**

|  | df | SS | MS | *F* | *R*^2^ | *p* |
| --- | --- | --- | --- | --- | --- | --- |
| Time | 5 | 13845 | 2768.9 | 8.73 | 0.1089 | 0.001 |
| Conc | 3 | 54748 | 18249.5 | 57.57 | 0.4308 | 0.001 |
| Time:Conc | 10 | 31208 | 3120.8 | 9.84 | 0.2456 | 0.001 |
| Residuals | 86 | 27259 | 317.0 |  | 0.2145 |  |
| Total | 104 | 127061 |  |  | 1.0000 |  |

Notes: all column labels correspond to those in Table S1.

For Eosinophils, both ‘Time’, ‘Conc’, and their interaction had a significant effect on ‘Area’ (all *p* < 0.050), with the effect size being slightly greater for ‘Conc’ (*R*2 = 0.3026) than for ‘Time’ (*R*2 = 0.2878) and its interaction with ‘Conc’ (*R*2 = 0.0622; **Table S6**). In this cell-type, the interaction seems to be much weaker than for Basophils, while the main effect of ‘Time’ seems much stronger than Basophils.

**Table S6. PERMANOVA testing the effect of time (‘Time’) and NaCl concentration (‘Conc’), and their interactions on Eosinophil cell size ('Area').**

|  | df | SS | MS | *F* | *R*^2^ | *p* |
| --- | --- | --- | --- | --- | --- | --- |
| Time | 5 | 30544 | 6108.9 | 14.58 | 0.2878 | 0.001 |
| Conc | 3 | 32122 | 10707.3 | 25.56 | 0.3026 | 0.001 |
| Time:Conc | 5 | 6604 | 1320.9 | 3.15 | 0.0622 | 0.013 |
| Residuals | 88 | 36859 | 418.8 |  | 0.3473 |  |
| Total | 101 | 106130 |  |  | 1.0000 |  |

Notes: all column labels correspond to those in Table S1.

For Lymphocytes, both ‘Time’, ‘Conc’, and their interaction had a significant effect on ‘Area’ (all *p* < 0.050), with the effect size being much greater for ‘Conc’ (*R*2 = 0.4697) than for ‘Time’ (*R*2 = 0.0239) and its interaction with ‘Conc’ (*R*2 = 0.0293; **Table S7**).

**Table S7. PERMANOVA testing the effect of time (‘Time’) and NaCl concentration (‘Conc’), and their interactions on Lymphocyte cell size ('Area').**

|  | df | SS | MS | *F* | *R*^2^ | *p* |
| --- | --- | --- | --- | --- | --- | --- |
| Time | 5 | 26213 | 5243 | 6.88 | 0.0239 | 0.001 |
| Conc | 3 | 514436 | 171479 | 225.19 | 0.4697 | 0.001 |
| Time:Conc | 13 | 32155 | 2473 | 3.24 | 0.0293 | 0.001 |
| Residuals | 686 | 522376 | 761 |  | 0.4769 |  |
| Total | 707 | 1095181 |  |  | 1.0000 |  |

Notes: all column labels correspond to those in Table S1.

For Monocytes, both ‘Time’, ‘Conc’, and their interaction had a significant effect on ‘Area’ (all *p* < 0.050), with the effect size being much greater for ‘Conc’ (*R*2 = 0.3021) than for ‘Time’ (*R*2 = 0.0694) and its interaction with ‘Conc’ (*R*2 = 0.1696; **Table S8**).

**Table S8. PERMANOVA testing the effect of time (‘Time’) and NaCl concentration (‘Conc’), and their interactions on Monocyte cell size ('Area').**

|  | df | SS | MS | *F* | *R*^2^ | *p* |
| --- | --- | --- | --- | --- | --- | --- |
| Time | 5 | 11716 | 2343.2 | 7.32 | 0.0694 | 0.001 |
| Conc | 3 | 50992 | 16997.4 | 53.11 | 0.3021 | 0.001 |
| Time:Conc | 13 | 28625 | 2201.9 | 6.88 | 0.1696 | 0.001 |
| Residuals | 242 | 77450 | 320.0 |  | 0.4588 |  |
| Total | 263 | 168783 |  |  | 1.0000 |  |

Notes: all column labels correspond to those in Table S1.

For Neutrophils, both ‘Time’, ‘Conc’, and their interaction had a significant effect on ‘Area’ (all *p* < 0.050), with the effect size being slightly greater for ‘Conc’ (*R*2 = 0.2769) than for ‘Time’ (*R*2 = 0.2054) and its interaction with ‘Conc’ (*R*2 = 0.0919; **Table S9**).

**Table S9. PERMANOVA testing the effect of time (‘Time’) and NaCl concentration (‘Conc’), and their interactions on Neutrophil cell size ('Area').**

|  | df | SS | MS | *F* | *R*^2^ | *p* |
| --- | --- | --- | --- | --- | --- | --- |
| Time | 5 | 20872 | 4174.4 | 20.56 | 0.2054 | 0.001 |
| Conc | 3 | 28135 | 9378.5 | 46.19 | 0.2769 | 0.001 |
| Time:Conc | 9 | 9340 | 1037.8 | 5.11 | 0.0919 | 0.001 |
| Residuals | 213 | 43245 | 203.0 |  | 0.4256 |  |
| Total | 230 | 101592 |  |  | 1.0000 |  |

Notes: all column labels correspond to those in Table S1.

Based on the *R*2 values, the effect of ‘Conc’ on ‘Area’ was greatest in Lymphocytes (*R*2 = 0.4697; **Table S7**), followed respectively by Basophils (*R*2 = 0.4308; **Table S5**), Eosinophils (*R*2 = 0.3026; **Table S6**), Monocytes (*R*2 = 0.3021; **Table S8**), and then Neutrophils (*R*2 = 0.2769; **Table S9**).

On the other hand, the effect of ‘Time’ on ‘Area’ was greatest in Eosinophils (*R*2 = 0.2878; **Table S6**), followed respectively by Neutrophils (*R*2 = 0.2054; **Table S9**), Basophils (*R*2 = 0.1089; **Table S5**), Monocytes (*R*2 = 0.0694; **Table S8**), and then Lymphocytes (*R*2 = 0.0239; **Table S7**).

A post hoc pairwise PERMANOVA done for each ‘CellType’ separately indicated a significant difference in the first neighboring ‘Conc’ pair (0.25 <-> 0.5 NaCl) for all WBC cell-types (all Holm-*p* < 0.050), with the effect being much greater for Neutrophils (*R*2 = 0.4035) than the other four cell-types (*R*2 ≤ 0.2572; **Table S10**). For the second neighboring ‘Conc’ pair (0.5 <-> 0.75 NaCl), the same post hoc pairwise PERMANOVA indicated no significant difference for all WBC cell-types (all Holm-*p* ≥ 0.134), except for Lymphocytes, where a significant difference was found, but of low effect (Holm-*p* < 0.050; *R*2 = 0.0816; **Table S10**). The same post hoc pairwise PERMANOVA indicated a significant difference in the third neighboring ‘Conc’ pair (0.75 <-> 0.9 NaCl) for all WBC cell-types (all Holm-*p* < 0.050), except for Neutrophils (Holm-*p* = 0.510; **Table S10**). Of the significant WBC cell-types, the effect size was greater in Eosinophils (*R*2 = 0.2841) and Basophils (*R*2 = 0.2371) than in Lymphocytes (*R*2 = 0.0809) and Monocytes (*R*2 = 0.0371; **Table S10**).

**Table S10. Pairwise PERMANOVAs testing the effect of NaCl concentration (‘Conc’) on cell size ('Area'), divided by each level of WBC cell-type (‘CellType’).**

| ‘Conc’ pair | SS | MS | *F* | *R*^2^ | *p* | Holm-*p* |
| --- | --- | --- | --- | --- | --- | --- |
| **Basophil** |  |  |  |  |  |  |
| *0.25 <-> 0.5* | *9328.6* | *9328.6* | *7.05* | *0.1194* | *0.010* | *0.020* |
| 0.25 <-> 0.75 | 13926.4 | 13926.4 | 11.56 | 0.1819 | 0.003 | 0.009 |
| 0.25 <-> 0.9 | 45607.0 | 45607.0 | 54.06 | 0.4465 | 0.001 | 0.006 |
| *0.5 <-> 0.75* | *344.3* | *344.3* | *0.50* | *0.0144* | *0.488* | *0.488* |
| 0.5 <-> 0.9 | 6479.1 | 6479.1 | 18.11 | 0.2699 | 0.001 | 0.006 |
| *0.75 <-> 0.9* | *3526.6* | *3526.6* | *15.22* | *0.2371* | *0.001* | *0.006* |
| **Eosinophil** |  |  |  |  |  |  |
| *0.25 <-> 0.5* | *4058.5* | *4058.5* | *7.77* | *0.1860* | *0.007* | *0.021* |
| 0.25 <-> 0.75 | 137.0 | 137.0 | 0.12 | 0.0025 | 0.726 | 0.726 |
| 0.25 <-> 0.9 | 17218.5 | 17218.5 | 84.55 | 0.6629 | 0.001 | 0.006 |
| *0.5 <-> 0.75* | *4268.8* | *4268.8* | *3.44* | *0.0589* | *0.067* | *0.134* |
| 0.5 <-> 0.9 | 4920.7 | 4920.7 | 10.75 | 0.1799 | 0.001 | 0.006 |
| *0.75 <-> 0.9* | *23503.3* | *23503.3* | *25.40* | *0.2841* | *0.001* | *0.006* |
| **Lymphocyte** |  |  |  |  |  |  |
| *0.25 <-> 0.5* | *176947.1* | *176947.1* | *119.83* | *0.2572* | *0.001* | *0.006* |
| 0.25 <-> 0.75 | 300419.8 | 300419.8 | 242.35 | 0.4119 | 0.001 | 0.006 |
| 0.25 <-> 0.9 | 439348.1 | 439348.1 | 401.63 | 0.5125 | 0.001 | 0.006 |
| *0.5 <-> 0.75* | *15196.7* | *15196.7* | *28.61* | *0.0816* | *0.001* | *0.006* |
| 0.5 <-> 0.9 | 45015.0 | 45015.0 | 100.71 | 0.2195 | 0.001 | 0.006 |
| *0.75 <-> 0.9* | *6868.4* | *6868.4* | *31.52* | *0.0809* | *0.001* | *0.006* |
| **Monocyte** |  |  |  |  |  |  |
| *0.25 <-> 0.5* | *11642.7* | *11642.7* | *14.65* | *0.1470* | *0.003* | *0.009* |
| 0.25 <-> 0.75 | 28216.6 | 28216.6 | 52.56 | 0.2977 | 0.001 | 0.006 |
| 0.25 <-> 0.9 | 45879.2 | 45879.2 | 135.50 | 0.4831 | 0.001 | 0.006 |
| *0.5 <-> 0.75* | *1483.8* | *1483.8* | *2.38* | *0.0203* | *0.136* | *0.136* |
| 0.5 <-> 0.9 | 5810.9 | 5810.9 | 14.56 | 0.0967 | 0.001 | 0.006 |
| *0.75 <-> 0.9* | *2051.4* | *2051.4* | *6.73* | *0.0371* | *0.007* | *0.014* |
| **Neutrophil** |  |  |  |  |  |  |
| *0.25 <-> 0.5* | *20617.0* | *20617.0* | *47.35* | *0.4035* | *0.001* | *0.006* |
| 0.25 <-> 0.75 | 23636.1 | 23636.1 | 101.19 | 0.5711 | 0.001 | 0.006 |
| 0.25 <-> 0.9 | 25295.1 | 25295.1 | 80.91 | 0.4707 | 0.001 | 0.006 |
| *0.5 <-> 0.75* | *579.3* | *579.3* | *1.67* | *0.0121* | *0.193* | *0.386* |
| 0.5 <-> 0.9 | 1309.0 | 1309.0 | 3.41 | 0.0221 | 0.055 | 0.165 |
| *0.75 <-> 0.9* | *128.2* | *128.2* | *0.45* | *0.0028* | *0.510* | *0.510* |

Notes: ‘Conc' pair’= pair of NaCl concentrations being compared; *p*= raw *p-*value based on 999 permutations; Holm-*p*= *p* value corrected for multiple comparisons using Holm's (1979) method. All other column labels correspond to those in Table S1. Neighboring ‘Conc’ pairs are in italics.

For the 0.25 NaCl concentration ‘Time’ did not have a significant effect on ‘Area’ (*p* = 0.507), while ‘CellType’ had a significant, but low effect (*p* < 0.050; *R*2 = 0.0781; **Table S11**).

**Table S11. PERMANOVA testing the effect of time (‘Time’) and WBC cell-type (‘CellType’), and their interactions on cell size ('Area') for 0.25 NaCl concentration.**

|  | df | SS | MS | *F* | *R*^2^ | *p* |
| --- | --- | --- | --- | --- | --- | --- |
| Time | 5 | 6283 | 1256.6 | 0.84 | 0.0122 | 0.507 |
| CellType | 4 | 40038 | 10009.6 | 6.74 | 0.0781 | 0.001 |
| Time:CellType | 14 | 69889 | 4992.1 | 3.36 | 0.1363 | 0.001 |
| Residuals | 267 | 396390 | 1484.6 |  | 0.7732 |  |
| Total | 290 | 512600 |  |  | 1.0000 |  |

Notes: all column labels correspond to those in Table S1.

For the 0.5 NaCl concentration, both ‘Time’, ‘CellType’, and their interaction had a significant effect on ‘Area’ (all *p* < 0.050), with the effect size being greatest for the interaction term (*R*2 = 0.1201; **Table S12**).

**Table S12. PERMANOVA testing the effect of time (‘Time’) and WBC cell-type (‘CellType’), and their interactions on cell size ('Area') for 0.5 NaCl concentration.**

|  | df | SS | MS | *F* | *R*^2^ | *p* |
| --- | --- | --- | --- | --- | --- | --- |
| Time | 4 | 15965 | 3991.4 | 6.37 | 0.0694 | 0.002 |
| CellType | 4 | 8516 | 2128.9 | 3.40 | 0.0370 | 0.015 |
| Time:CellType | 13 | 27624 | 2124.9 | 3.39 | 0.1201 | 0.001 |
| Residuals | 284 | 177768 | 625.9 |  | 0.7733 |  |
| Total | 305 | 229873 |  |  | 1.0000 |  |

Notes: all column labels correspond to those in Table S1.

For the 0.75 NaCl concentration, both ‘Time’, ‘CellType’, and their interaction had a significant effect on ‘Area’ (all *p* < 0.050), with the effect size being much greater for ‘Time’ (*R*2 = 0.3453) than for ‘CellType’ (*R*2 = 0.0962) and their interaction (*R*2 = 0.1083; **Table S13**).

**Table S13. PERMANOVA testing the effect of time (‘Time’) and WBC cell-type (‘CellType’), and their interactions on cell size ('Area') for 0.75 NaCl concentration.**

|  | df | SS | MS | *F* | *R*^2^ | *p* |
| --- | --- | --- | --- | --- | --- | --- |
| Time | 4 | 66698 | 16674.5 | 65.97 | 0.3453 | 0.001 |
| CellType | 4 | 18587 | 4646.7 | 18.38 | 0.0962 | 0.001 |
| Time:CellType | 13 | 20918 | 1609.1 | 6.36 | 0.1083 | 0.001 |
| Residuals | 344 | 86941 | 252.7 |  | 0.4501 |  |
| Total | 365 | 193144 |  |  | 1.0000 |  |

Notes: all column labels correspond to those in Table S1.

For the 0.9 NaCl concentration, both ‘Time’, ‘CellType’, and their interaction had a significant effect on ‘Area’ (all *p* < 0.050), with the effect size being much greater for ‘Time’ (*R*2 = 0.3981) than for ‘CellType’ (*R*2 = 0.0164) and their interaction (*R*2 = 0.0804; **Table S14**).

**Table S14. PERMANOVA testing the effect of time (‘Time’) and WBC cell-type (‘CellType’), and their interactions on cell size ('Area') for 0.9 NaCl concentration.**

|  | df | SS | MS | *F* | *R*^2^ | *p* |
| --- | --- | --- | --- | --- | --- | --- |
| Time | 5 | 36329 | 7265.9 | 66.21 | 0.3981 | 0.001 |
| CellType | 4 | 1499 | 374.8 | 3.41 | 0.0164 | 0.006 |
| Time:CellType | 17 | 7337 | 431.6 | 3.93 | 0.0804 | 0.001 |
| Residuals | 420 | 46090 | 109.7 |  | 0.5050 |  |
| Total | 446 | 91256 |  |  | 1.0000 |  |

Notes: all column labels correspond to those in Table S1.

Based on the *R*2 values, the effect of ‘CellType’ on ‘Area’ was generally low across NaCl concentrations (*R*2 = 0.0164–0.0962; **Tables S11-14**). On the other hand, the effect of ‘Time’ on ‘Area’ was much greater for the 0.9 NaCl (*R*2 = 0.3981; **Table S14**) and the 0.75 NaCl (*R*2 = 0.3453; **Table S13**) concentrations than in the 0.25 NaCl (*R*2 = 0.0122; **Table S11**) and the 0.5 NaCl (*R*2 = 0.0694; **Table S12**) concentrations.

A post hoc pairwise PERMANOVA done for each ‘Conc’ separately indicated that for the 0.25 NaCl concentration, only three pairs exhibited significant differences: Eosinophil vs. Neutrophil, Lymphocyte vs. Monocyte, and Monocyte vs. Neutrophil (all Holm-*p* < 0.050), with the effect size being much greater for Eosinophil vs. Neutrophil (*R*2 = 0.5734) than the other two pairs (*R*2 = 0.0696–0.1607; **Table S15**). The same post hoc pairwise PERMANOVA indicated no significant differences among any pair for the 0.5 NaCl concentration (**Table S15**). The same post hoc pairwise PERMANOVA indicated that for the 0.75 NaCl concentration, only three pairs exhibited significant differences: Eosinophil vs. each of Lymphocyte, Monocyte, and Neutrophil (all Holm-*p* < 0.050), with the effect size being similar for each pair (*R*2 = 0.2311–0.2790; **Table S15**). The same post hoc pairwise PERMANOVA indicated that for the 0.9 NaCl concentration, only one pair (Lymphocyte vs. Neutrophil) exhibited a significant difference (Holm-*p* < 0.050), with a very low effect size (*R*2 = 0.0312; **Table S15**).

**Table S15. Pairwise PERMANOVAs testing the effect of WBC cell-type (‘CellType’) on cell size ('Area'), divided by each level of NaCl concentration (‘Conc’).**

| ‘CellType’ pair | SS | MS | F | R^2^ | p | Holm-p |
| --- | --- | --- | --- | --- | --- | --- |
| **0.25 NaCl** |  |  |  |  |  |  |
| Basophil <-> Eosinophil | 214.7 | 214.7 | 0.19 | 0.0038 | 0.680 | 1.000 |
| Basophil <-> Lymphocyte | 10350.3 | 10350.3 | 5.20 | 0.0231 | 0.019 | 0.133 |
| Basophil <-> Monocyte | 2069.6 | 2069.6 | 1.99 | 0.0237 | 0.162 | 0.810 |
| Basophil <-> Neutrophil | 2886.9 | 2886.9 | 2.12 | 0.0504 | 0.165 | 0.810 |
| Eosinophil <-> Lymphocyte | 7360.3 | 7360.3 | 3.79 | 0.0187 | 0.056 | 0.336 |
| Eosinophil <-> Monocyte | 349.1 | 349.1 | 0.64 | 0.0104 | 0.452 | 1.000 |
| Eosinophil <-> Neutrophil | 3407.2 | 3407.2 | 25.54 | 0.5734 | 0.002 | 0.018 |
| Lymphocyte <-> Monocyte | 31109.3 | 31109.3 | 17.37 | 0.0696 | 0.001 | 0.010 |
| Lymphocyte <-> Neutrophil | 155.3 | 155.3 | 0.08 | 0.0004 | 0.783 | 1.000 |
| Monocyte <-> Neutrophil | 6065.3 | 6065.3 | 9.96 | 0.1607 | 0.002 | 0.018 |
| **0.5 NaCl** |  |  |  |  |  |  |
| Basophil <-> Eosinophil | 32.9 | 32.9 | 0.04 | 0.0011 | 0.831 | 1.000 |
| Basophil <-> Lymphocyte | 23.3 | 23.3 | 0.03 | 0.0002 | 0.868 | 1.000 |
| Basophil <-> Monocyte | 360.4 | 360.4 | 0.39 | 0.0070 | 0.530 | 1.000 |
| Basophil <-> Neutrophil | 1317.3 | 1317.3 | 2.41 | 0.0285 | 0.132 | 1.000 |
| Eosinophil <-> Lymphocyte | 7.7 | 7.7 | 0.01 | 0.0001 | 0.919 | 1.000 |
| Eosinophil <-> Monocyte | 717.8 | 717.8 | 0.80 | 0.0136 | 0.372 | 1.000 |
| Eosinophil <-> Neutrophil | 2104.3 | 2104.3 | 3.91 | 0.0440 | 0.059 | 0.531 |
| Lymphocyte <-> Monocyte | 1373.2 | 1373.2 | 1.68 | 0.0084 | 0.204 | 1.000 |
| Lymphocyte <-> Neutrophil | 5521.4 | 5521.4 | 7.97 | 0.0341 | 0.009 | 0.090 |
| Monocyte <-> Neutrophil | 440.9 | 440.9 | 0.68 | 0.0066 | 0.417 | 1.000 |
| **0.75 NaCl** |  |  |  |  |  |  |
| Basophil <-> Eosinophil | 8091.6 | 8091.6 | 6.87 | 0.1167 | 0.018 | 0.126 |
| Basophil <-> Lymphocyte | 645.5 | 645.5 | 2.16 | 0.0120 | 0.130 | 0.650 |
| Basophil <-> Monocyte | 672.1 | 672.1 | 1.43 | 0.0150 | 0.225 | 0.900 |
| Basophil <-> Neutrophil | 824.7 | 824.7 | 2.79 | 0.0308 | 0.104 | 0.624 |
| Eosinophil <-> Lymphocyte | 30690.6 | 30690.6 | 62.01 | 0.2403 | 0.001 | 0.010 |
| Eosinophil <-> Monocyte | 26413.0 | 26413.0 | 33.66 | 0.2311 | 0.001 | 0.010 |
| Eosinophil <-> Neutrophil | 26990.1 | 26990.1 | 41.01 | 0.2790 | 0.001 | 0.010 |
| Lymphocyte <-> Monocyte | 11.5 | 11.5 | 0.03 | 0.0001 | 0.858 | 1.000 |
| Lymphocyte <-> Neutrophil | 78.6 | 78.6 | 0.30 | 0.0013 | 0.578 | 1.000 |
| Monocyte <-> Neutrophil | 23.3 | 23.3 | 0.07 | 0.0004 | 0.790 | 1.000 |
| **0.9 NaCl** |  |  |  |  |  |  |
| Basophil <-> Eosinophil | 470.1 | 470.1 | 3.07 | 0.0479 | 0.077 | 0.539 |
| Basophil <-> Lymphocyte | 150.8 | 150.8 | 0.96 | 0.0042 | 0.310 | 1.000 |
| Basophil <-> Monocyte | 350.7 | 350.7 | 2.22 | 0.0168 | 0.141 | 0.846 |
| Basophil <-> Neutrophil | 1542.3 | 1542.3 | 5.95 | 0.0480 | 0.014 | 0.126 |
| Eosinophil <-> Lymphocyte | 260.3 | 260.3 | 1.46 | 0.0064 | 0.222 | 1.000 |
| Eosinophil <-> Monocyte | 66.9 | 66.9 | 0.35 | 0.0027 | 0.588 | 1.000 |
| Eosinophil <-> Neutrophil | 146.2 | 146.2 | 0.48 | 0.0042 | 0.485 | 1.000 |
| Lymphocyte <-> Monocyte | 139.9 | 139.9 | 0.80 | 0.0027 | 0.376 | 1.000 |
| Lymphocyte <-> Neutrophil | 1977.8 | 1977.8 | 9.11 | 0.0312 | 0.004 | 0.040 |
| Monocyte <-> Neutrophil | 842.2 | 842.2 | 3.38 | 0.0180 | 0.065 | 0.520 |

Notes: ‘Conc' pair’= pair of NaCl concentrations being compared; *p*= raw *p-*value based on 999 permutations; Holm-*p*= *p* value corrected for multiple comparisons using Holm's (1979) method. All other column labels correspond to those in Table S1. Neighboring ‘Conc’ pairs are in italics.
